# DIFENSE Study Protocol: Early Intervention With Difamilast Ointment in Infantile Early‐Onset Atopic Dermatitis for Prevention of Transcutaneous Sensitisation

**DOI:** 10.1111/cea.70039

**Published:** 2025-03-26

**Authors:** Kiwako Yamamoto‐Hanada, Kazuyoshi Okamoto, Nobuharu Kishimoto, Kunihiko Tsuchiya, Osamu Natsume, Yutaka Takemura, Masami Narita, Tohru Kobayashi, Yukihiro Ohya

**Affiliations:** ^1^ Allergy Center National Center for Child Health and Development Tokyo Japan; ^2^ Otsuka Pharmaceutical Co., Ltd. Tokyo Japan; ^3^ Department of Pediatrics Graduate School of Medical Science, Kyoto Prefectural University of Medicine Kyoto Japan; ^4^ Department of Pediatrics Hamamatsu University School of Medicine Shizuoka Japan; ^5^ Department of Pediatrics Kindai University, Faculty of Medicine Osaka Japan; ^6^ Department of Pediatrics Kyorin University School of Medicine Tokyo Japan; ^7^ Division of Public Health Jichi Medical University Tochigi Japan; ^8^ Department of Occupational and Environmental Health Graduate School of Medical Sciences, Nagoya City University Aichi Japan; ^9^ Division of General Allergy Bantane Hospital, Fujita Health University Aichi Japan

**Keywords:** atopic dermatitis, difamilast, food allergy, prevention, proactive therapy, sensitisation

**Summary**

- The DIFENSE Study is a multicentre randomised trial on early difamilast use for food allergy prevention.
- This study aimed to provide key evidence supporting skin intervention to prevent percutaneous sensitisation.

**Abbreviations**
ad
: atopic dermatitis
FA: food allergy
RCT: randomised controlled trial

To the editor,

Atopic dermatitis (ad) is acknowledged as the first phase in the allergic march, with heightened IgE levels [1] linked to a greater likelihood of experiencing food allergy symptoms [2]. Importantly, the occurrence of early‐onset ad during infancy has been found to significantly increase the odds ratio for the development of food allergies [3]. Additionally, suitable transdermal and oral interventions during infancy may impact the trajectory of the allergic march, which includes the prevention of food allergies. The ‘dual allergen exposure hypothesis’ has been suggested as a mechanistic framework for the development of allergies, and since randomised controlled trials (RCTs) have validated the effectiveness of both transdermal and oral treatments, this hypothesis has effectively evolved into an established theory.

Numerous RCTs have explored the potential of moisturisers in preventing food allergies as part of skincare strategies. Nevertheless, variations in study populations, types of moisturisers, frequencies of application and skin cleansing protocols have led to inconsistent results. Large‐scale RCTs carried out in Northern Europe and the United Kingdom have not substantiated the preventative effects of moisturisers on food allergies. A Cochrane Review by Kelleher et al. [4] determined that skincare interventions, which include the use of moisturisers, do not aid in the prevention of food allergies in children.

ad is distinguished by dysfunctional skin barrier, chronic inflammation and pruritus. Interventions that focus exclusively on the restoration of the skin barrier through moisturisers have been demonstrated to be insufficient in mitigating the onset of food allergies. In addition, a RCT performed demonstrated that the utilisation of a mild nonsteroidal topical agent (pimecrolimus) solely on affected eczema lesions did not inhibit the development of food allergies [5]. This outcome indicates that minimal anti‐inflammatory treatments targeting only visibly affected eczema regions may fall short in reducing the risk of food allergies. Importantly, even skin that appears normal shows compromised barrier function and heightened inflammatory markers, reflecting the existence of subclinical inflammation at the molecular level [6]. As a result, successfully preventing percutaneous sensitisation demands a well‐rounded strategy that integrates barrier improvement and anti‐inflammatory interventions, reaching beyond visible lesions to cover nonlesional skin.

**FIGURE 1:**
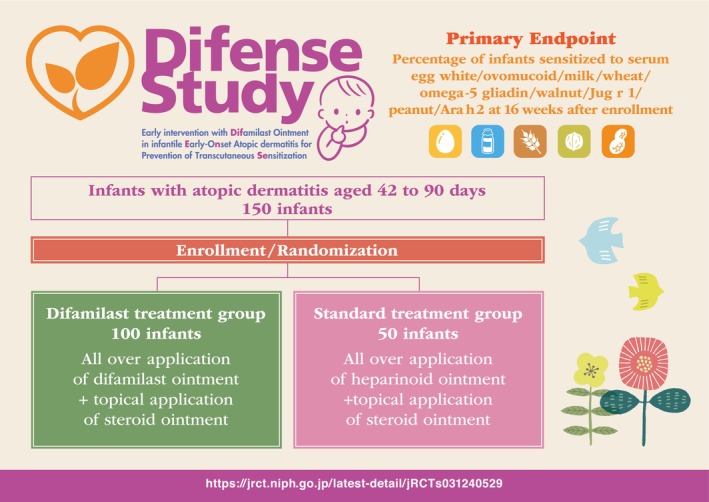
The synopsis of the DIFENSE Study.

Infants who manifest eczema during the first 1–2 months of life exhibit the greatest likelihood of developing food allergies, particularly those exhibiting eczema before the introduction of solid foods, which exacerbates their risk further [3]. As a result, infants with early‐onset ad are the demographic that most urgently needs preventive actions. A retrospective analysis conducted at our institution indicated that prompt intervention following the manifestation of ad, encompassing proactive treatment administered to nonlesional regions, was associated with a reduced probability of developing food allergies [7]. After achieving remission, maintaining strict control of eczema and preventing percutaneous sensitisation becomes essential to lower the risk of IgE‐mediated food allergies.

Conventional treatment strategies that concentrate entirely on visibly affected lesions might not be sufficient for attaining complete control of inflammation [5]. In the PACI Study [8], a multicentre randomised controlled trial conducted in 2023, a proactive early treatment protocol was implemented, which included applying topical corticosteroids (TCS) not only to the visibly affected eczematous lesions but also to nonlesional skin areas. This intervention produced a 25% decline in the prevalence of egg allergy by the age of 28 weeks relative to standard treatment. However, the intervention group statistically significantly lowered body weight and height compared with the conventional group, although a definitive link between TCS dosage and these outcomes (weight and height) was not found. These results suggest the importance of individualised treatment plans rather than a generalised approach for all infants.

Recently, difamilast ointment [9], a new nonsteroidal topical medication, has come to light as an adjunctive treatment alongside TCS. Difamilast supports the implementation of a proactive treatment, allowing persistent inflammation control while alleviating adverse effects, particularly during the period of remission maintenance. To delve deeper into the effectiveness of early intervention with difamilast ointment in preventing percutaneous sensitisation in infants affected by early‐onset atopic dermatitis (ad), we have embarked on the DIFENSE Study (jRCTs031240529) (Figure 1).

The DIFENSE Study (Early Intervention with Difamilast Ointment in Infantile Early‐Onset Atopic Dermatitis for Prevention of Transcutaneous Sensitization) is a multicentre, randomised, comparative and exploratory trial that will last for 16 weeks. The study plans to recruit 150 infants, with 100 allocated to the difamilast treatment group and 50 to the standard treatment group. The primary endpoint is to assess the percentage of infants sensitised to serum egg white, ovomucoid, milk, wheat, omega‐5 gliadin, walnut, Jug r 1, peanut and Ara h 2 at 16 weeks following enrolment. Eligible participants include infants aged between 42 and 90 days who have been diagnosed with ad based on UKWP criteria within 28 days of the onset of their initial symptoms. The study protocol is in this article's Online Repository at https://zenodo.org/records/14942018.

We anticipate that this study will provide pioneering evidence in support of advanced strategies designed to prevent percutaneous sensitisation and the subsequent onset of food allergies.

## Author Contributions

K.Y.‐H., K.O., N.K., T.K. and Y.O. conceived the idea of the study. All authors contributed to establishing the study protocol. Y.O. supervised the conduct of this study. All authors reviewed the manuscript draft and revised it critically for intellectual content. All authors approved the publication of the final version of the manuscript.

## Conflicts of Interest

The National Center for Child Health and Development and Otsuka Pharmaceutical Co. Ltd. signed a joint research and development agreement. K.O. and N.K. are employees of Otsuka Pharmaceutical Co. Ltd.

## Data Availability

Currently, individual participant data sharing is unavailable because the IRB permission is not yet obtained. Individual participant data sharing will be available after the IRB permission is granted. Any data queries can be emailed to the Primary Investigator Kiwako Yamamoto‐Hanada at yamamoto-k@ncchd.go.jp.
